# Comparative Evaluation of Microbial Colonization in Spot-Welded and Laser-Welded Orthodontic Bands: A Quantitative Analysis

**DOI:** 10.7759/cureus.109899

**Published:** 2026-05-29

**Authors:** Sandra V Kala Vani, Firoz Babu, Vijaysinh R Tanpure, Bindu S Priya, Hemadri Sreerangam, Pavan Sai

**Affiliations:** 1 Department of Orthodontics, Chadalawada Krishna Srinivasa (CKS) Teja Institute of Dental Sciences and Research, Tirupati, IND; 2 Department of Orthodontics, Yashwantrao Chavan Dental College, Ahilyanagar, IND; 3 Department of Orthodontics, Dollar Smiles Dental Hospital, Hyderabad, IND

**Keywords:** bacterial load, dental plaque, oral hygiene, orthodontic appliances, welding

## Abstract

Objective

The present study quantitatively evaluated and compared bacterial counts between spot-welded and laser-welded orthodontic bands. The need for the study arose from concerns that conventional spot-welded orthodontic bands may create irregular surfaces and microgaps that favor plaque accumulation and bacterial colonization, thereby increasing the risk of enamel decalcification and periodontal inflammation during orthodontic treatment. Laser welding has been proposed as an alternative technique capable of producing smoother and more precise joints with potentially reduced bacterial retention. However, limited clinical evidence is available comparing bacterial colonization associated with these two welding techniques. Therefore, this study was undertaken to assess and compare bacterial counts on spot-welded and laser-welded orthodontic bands. To address this objective, the following methodology was implemented.

Methodology

The study employed a split-mouth design with 10 subjects requiring orthodontic treatment. Laser-welded bands and spot-welded bands on the right and left sides of the mandible were cemented, which represent the experimental and control sides, respectively. Plaque was collected from buccal and lingual surfaces on both control and experimental sides at baseline and after one month by a single examiner to prevent inter-individual bias. Plaque was diluted by serial dilution and then inoculated onto a culture plate containing agar as the growth medium. After three days, the number of bacterial colonies was counted using a digital colony counter, yielding colony-forming units (CFUs).

Results

The study showed that bacterial counts increased significantly on both the spot-welded and laser-welded sides after band placement on the buccal and lingual aspects. The bacterial count was higher on the spot-welded side than on the laser-welded side, but the difference was not statistically significant (p>0.05).

Conclusions

Based on the study, there was no significant microbiological difference between laser-welded and conventional spot-welded orthodontic bands. But the presence of orthodontic bands, in the absence of optimal oral hygiene, will promote plaque growth. So, patients should be educated and motivated to maintain good oral hygiene.

## Introduction

There are more than 600 bacterial species that comprise the plaque microflora on the surfaces of the oral cavity. Changes in environmental conditions within the oral cavity can alter the microflora. There is an increased prevalence of oral diseases, including dental caries, gingivitis, and periodontitis, when orthodontic appliances are attached to tooth enamel [1-3]. With growing awareness of esthetics among the general population, there is a strong demand for active orthodontic treatment, which usually lasts a year or slightly longer [4].

Fixed orthodontic appliances are considered highly effective for starting active treatment. However, they make tooth cleaning difficult and favour plaque formation. These appliances also restrict the self-cleansing action of the tongue, lips, and cheeks, thereby reducing the removal of food debris. Most microbial growth sites are found at the gingival margin and on the edges of orthodontic bands [5,6].A lack of adequate oral hygiene, combined with fixed orthodontic appliances, leads to increased bacterial plaque and an increased inflammatory response [7].

The drawback of these fixed attachments is the accumulation and retention of plaque. This increases the risk of white spot lesions and enamel demineralisation if the patient does not maintain oral hygiene or use proper aids [8]. The presence of orthodontic bands as foreign bodies can increase pathogenic bacterial levels and increase the risk of caries and infections if oral hygiene is not maintained.

Either spot welding, laser welding, pressure welding, or oxyacetylene flame welding can be used to weld orthodontic bands. Among these methods, spot welding and laser welding are most commonly employed [9]. A brief overview of the mechanics and implications of each method follows.

Spot-welding joins two or more surfaces through heat generated by an electric current. Force is applied by two electrodes holding the pieces together. Laser welding joins surfaces by localised fusion using a beam of concentrated, coherent, monochromatic high-intensity light. Different laser sources include Nd: YAG, CO2, AlGaAs, and Yb-doped fibre lasers. Nd: YAG lasers are widely used. Laser welding eliminates distortion, porosities, and corrosion [10-12].

Several clinical and microbiological studies indicate that, without proper oral hygiene, orthodontic bands can increase microbial counts and alter the quality of the microbial community [13]. No previous studies have assessed bacterial counts with laser-welded bands, either qualitatively or quantitatively. This study aims to quantify and compare bacterial counts for spot-welded and laser-welded bands. The null hypothesis states that there would be no significant difference in bacterial colonization between laser-welded and spot-welded orthodontic bands after one month of intraoral use.

## Materials and methods

Study sample

The sample comprised 10 patients who required fixed appliance therapy for their existing malocclusion. The sample size was selected based on the feasibility of the split-mouth study design, where each patient served as their own control, thereby reducing inter-individual variability and improving comparison reliability. The sample size was considered adequate for preliminary quantitative evaluation of bacterial colonization between spot-welded and laser-welded orthodontic bands during the one-month study period.

Inclusion criteria

Patients requiring fixed orthodontic appliance therapy were included in the study. Only patients with fully erupted, bilaterally permanent mandibular first molars and good general and oral health were selected. Patients with no active periodontal disease or dental caries affecting the mandibular first molars, adequate oral hygiene maintenance, and willingness to participate by providing informed consent were included in the study.

Exclusion criteria

Patients with systemic diseases affecting oral health or salivary flow were excluded from the study. Individuals who had undergone antibiotic therapy or used antimicrobial mouth rinses within the previous three months were also excluded. Patients presenting with poor oral hygiene, active gingival or periodontal disease, temporomandibular joint dysfunction, extensively restored, carious, or malformed mandibular first molars, and those with previous orthodontic treatment involving molar banding were not included. Smokers, tobacco users, and patients unwilling to participate or comply with the study protocol were also excluded from the study.

The study details were explained to the participants, and written informed consent was obtained from all participants prior to commencement of the study. The study was conducted in accordance with the principles of the Declaration of Helsinki for biomedical research. The Ethical Committee of the C.K.S. Teja Institute of Dental Sciences & Research, Tirupati, approved it (CKS/Ortho/SS/24/01).

Method

The study period was one month, with plaque serving as the sample for bacterial count. A split-mouth design was used to compare bacterial counts. Mandibular first molars were selected because they are routinely banded teeth with high plaque-retentive potential, similar bilateral functional conditions, and provide standardized sites for reliable split-mouth comparison of bacterial colonization. Spot-welded and laser-welded bands were cemented on mandibular first molars, with laser-welded bands on the right side and spot-welded bands on the left side serving as the experimental and control sides, respectively.

Clinical procedure

Plaque samples were collected from the buccal and lingual aspects of the lower first molars on both the control and experimental sides at baseline and at the end of the study period (i.e., after one month) by a single examiner to prevent inter-individual bias. After an initial plaque collection at baseline, bands with attachments were cemented to the lower first molars on the left (Spot-welded) and right (Laser-welded) sides, constituting the control and experimental sides, respectively. No other attachments were placed in the adjacent areas that would alter the bacterial load at the experimental or control sites.

Plaque collection procedure

The patients were seated upright in the dental chair under adequate illumination. Prior to plaque collection, the operator isolated the mandibular first molars using sterile cotton rolls and gently air-dried the area to minimize salivary contamination. A sterile mouth mirror and sterile gutta-percha points were used for plaque sampling. Plaque samples were collected from four standardized sites: the buccal and lingual aspects of tooth 36 (control side - spot-welded band) and the buccal and lingual aspects of tooth 46 (experimental side - laser-welded band). The samples were designated as A, B, C, and D corresponding to the buccal and lingual aspects of teeth 36 and 46, respectively.

The sterile gutta-percha point was inserted gently along the gingival margin adjacent to the orthodontic band and maintained in contact with the plaque biofilm for approximately 10 seconds. Immediately after collection, each sample was transferred aseptically into a sterile 2 mL Eppendorf tube containing 1 mL sterile normal saline solution (0.9% NaCl). Gutta-percha points contaminated with saliva or blood during sampling were discarded and replaced with new sterile points to avoid sample contamination.

To minimize procedural variability, all plaque samples were collected by a single calibrated examiner using the same sampling protocol throughout the study period. Prior to commencement of the study, examiner calibration was performed on five non-study patients under supervision to standardize plaque collection technique, sample handling, and transport procedures.

Orthodontic band preparation and cementation procedure

Preformed stainless steel mandibular first molar bands were adapted and fitted clinically to achieve optimal marginal adaptation. Spot-welded bands were used on the left mandibular first molars (36), while laser-welded bands were placed on the right mandibular first molars (46). Before cementation, all bands were cleaned with alcohol and air-dried.

Band cementation was carried out using conventional Type I glass ionomer luting cement (GC Fuji I®, GC Corporation, Tokyo, Japan) according to the manufacturer’s instructions. The tooth surfaces were cleaned with pumice, rinsed thoroughly, and dried before band placement. Excess cement around the band margins was carefully removed after seating to minimize plaque-retentive areas.

Patient instructions

Following initial plaque removal and orthodontic band cementation, all patients received standardized oral hygiene instructions. Patients were instructed to continue their routine oral hygiene measures, including brushing twice daily for at least 2 minutes using fluoridated toothpaste. No additional antimicrobial mouth rinses or adjunctive oral hygiene aids were prescribed during the study period. Patients were advised to avoid antibiotic medications unless medically necessary and to report any medication usage during the study duration. The patients were recalled after one month for follow-up plaque sample collection.

Evaluation of bacterial count

To minimize observer bias, microbiological processing and colony counting were performed by an investigator who was blinded to the allocation of spot-welded and laser-welded samples during bacterial enumeration and colony-forming unit (CFU) analysis. Immediately after sample collection, the plaque samples were vortex mixed for 30 seconds to obtain a homogeneous bacterial suspension. Serial tenfold dilutions were prepared aseptically using sterile normal saline as the diluent. From the original suspension, 0.1 mL of sample was transferred into 0.9 mL sterile saline to obtain a 10⁻¹ dilution. This procedure was repeated sequentially until a final dilution factor of 10⁻⁶ was achieved.

Viable bacterial counts were determined using the pour plate technique. For each selected dilution, 0.1 mL of diluted sample was inoculated into sterile Petri dishes, followed by addition of approximately 15-20 mL of molten Brain Heart Infusion (BHI) agar maintained at 45-50°C. The inoculum and agar were mixed gently by circular rotation of the plate to ensure uniform distribution before solidification.

Brain Heart Infusion agar (HiMedia Laboratories Pvt. Ltd., Mumbai, India) was used as the culture medium. The medium composition included calf brain infusion, beef heart infusion, proteose peptone, dextrose, sodium chloride, disodium phosphate, and agar as specified by the manufacturer.

The inoculated plates were incubated under anaerobic conditions in an anaerobic chamber containing 5% CO₂, 10% H₂, and 85% N₂ at 37°C for 48 hours. Following incubation, plates demonstrating discrete and countable colonies were selected for analysis. Plates containing 30-300 colonies were considered acceptable for accurate bacterial enumeration.

The total number of colony-forming units (CFUs) was counted manually using a digital colony counter, and the bacterial count was calculated using the formula:

CFU/mL = Number of colonies × Dilution factor / Volume plated

The bacterial counts obtained were expressed as colony-forming units per milliliter (CFU/mL). Subsequently, microbial cultures were harvested and suspended in Brain Heart Infusion broth adjusted to a 4.0 McFarland turbidity standard measured at 660 nm for standardization of bacterial concentration [14].

## Results

**Figure 1:**
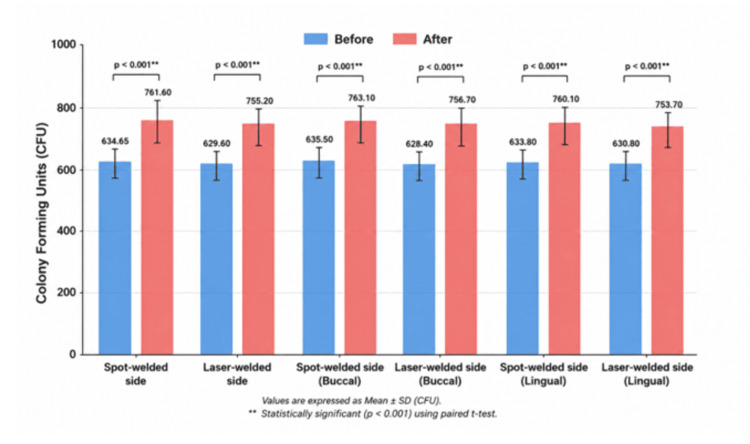
Intergroup comparison of colony-forming units (CFUs)

Because of the split-mouth study design, paired statistical analysis was performed for both intragroup and intergroup comparisons. Paired t-tests were used to compare bacterial counts between baseline and one month, as well as between spot-welded and laser-welded sides. Statistical analysis was performed using SPSS version 23 (IBM Corp., Armonk, NY, USA), and p < 0.05 was considered statistically significant. Using information obtained at two time points (baseline and day 30), the study results are reported in Figure 1.

Intragroup comparisons

Among spot-welded and laser-welded sides, a statistically significant (p<0.05) increase in CFUs was seen after one month of band placement. A similar increase was observed on both the buccal and lingual aspects of the spot- and laser-welded sides.

Intergroup comparisons

An intergroup comparison of spot- and laser-welded sides showed no statistically significant difference (p>0.05) in CFUs before or after one month of band placement. No significant difference was observed on the buccal or lingual aspect.

When buccal vs lingual aspects of both spot and laser-welded sides were compared, statistically insignificant (p>0.05) differences in colony-forming units were observed on both buccal and lingual aspects.

## Discussion

Local factors can contribute to plaque buildup and the development of gingivitis. Clinical observations have shown that elements such as irregularly aligned teeth, faulty restorations, fixed crowns, removable prosthetic or orthodontic appliances, and orthodontic bands are often linked to gingival inflammation [15]. Fixed orthodontic appliances make it difficult to maintain good oral hygiene and promote plaque retention, harbouring harmful microflora [5].

Over time, there is greater microbial colonisation around orthodontic attachments. This may be attributed to orthodontic attachments serving as a retention mechanism for dental plaque. Alterations in the physical and chemical characteristics of the environment resulting from the presence of bands may favour the growth of opportunistic periodontal pathogens or bacteria associated with caries, altogether increasing the bacterial load [15].

Several authors have reported deterioration in gingival health during orthodontic treatment with fixed appliances: Baer and Coccaro (1964) [16], Pearson (1968) [17], Rateitschak et al. (1968) [18], Zachrisson and Alnaes (1973) [19], and Kloehn and Pfeifer (1974) [20]. The increase in pathogenicity of the dental plaque and the concomitant periodontal changes during orthodontic treatment have been described by several authors: Petti et al. (1997) [21], Naranjo et al. (2006) [22], Gastel et al. (2008) [23], Sallum et al. [24]. This may, at least in part, be attributed to mechanical injury caused by the subgingival placement of the orthodontic bands [25]. On the other hand, the direct influence of slightly subgingivally placed bands on plaque accumulation rate and composition cannot be excluded [26].

The rise in plaque scores on banded teeth aligns with other research. Zachrisson [27], Huser et al. (1990) [15], Boyd and Baumrind (1992) [28], Levrini (2013) [29], and Al-Anezi (2015) [30] have noted increased microbial biofilm and plaque accumulation during orthodontic treatment.

To date, many qualitative studies have evaluated the growth of various microorganisms associated with orthodontic attachments, compared bands and brackets, and examined microbes associated with supra vs. subgingival plaque, among other topics. However, studies reporting the microbiological aspects of spot- and laser-welded orthodontic bands were lacking.

From the results, it was shown that bacterial count increased significantly (p < 0.05) for both experimental and control teeth, i.e., spot-welded and, on the laser-welded side, after band placement during the study period, on both the buccal and lingual aspects. Results revealed that spot-welded bands had higher bacterial counts than laser-welded bands, but the difference was not statistically significant (p>0.05). When buccal and lingual aspects were compared, the bacterial count was higher on the buccal aspect than the lingual aspect for both experimental and control teeth. Still, this difference was not statistically significant (p>0.05). Total CFU quantification reflects only the overall bacterial load. The absence of significant differences in total CFU counts does not necessarily exclude the possibility of clinically relevant compositional microbial changes or selective enrichment of pathogenic bacterial species between the two welding methods. These insignificant values may be attributable to a small sample size (n=10) and a short study period of one month.

The present investigation should be considered a pilot exploratory clinical study intended to provide preliminary microbiological data regarding bacterial colonization associated with different orthodontic band welding techniques. The one-month observation period represents an early-phase evaluation of bacterial colonization and may not fully reflect long-term orthodontic biofilm maturation, enamel demineralization potential, or periodontal alterations associated with orthodontic banding. Longer longitudinal studies are required to evaluate sustained microbiological and clinical changes. Although standardized oral hygiene instructions were provided to all participants, patient compliance with oral hygiene measures was not objectively monitored using plaque index, gingival index, or compliance scoring systems. Variations in individual oral hygiene practices may therefore have influenced bacterial colonization patterns and constituted a potential confounding factor.

The present study evaluated only total cultivable bacterial counts using non-selective Brain Heart Infusion agar and did not perform species-specific microbial identification or molecular microbiological analysis. Therefore, selective colonization by cariogenic or periodontal pathogens such as Streptococcus mutans, Lactobacillus spp., or anaerobic periodontal microorganisms could not be assessed. Despite these limitations, the split-mouth design minimized inter-individual variability and allowed standardized comparison between the two welding techniques under similar intraoral conditions. Future studies employing selective culture media, PCR-based techniques, or next-generation sequencing methods are recommended for comprehensive microbial characterization.

## Conclusions

Within the limitations of this pilot clinical study, both spot-welded and laser-welded orthodontic bands demonstrated increased bacterial colonization after one month of intraoral use. Although laser-welded bands showed comparatively lower bacterial counts, the differences were not statistically significant. Due to the limited sample size, short follow-up duration, and absence of species-specific microbiological analysis, these findings should be interpreted as preliminary and exploratory rather than definitive. Hence, no recommendations regarding the welding process could be made based on the study.

Looking into the future, more randomised controlled prospective clinical trials involving larger numbers of subjects over longer durations, incorporating molecular microbiological techniques and species-level microbial analysis, should be carried out to enable more comprehensive evaluation of orthodontic band-associated biofilm changes.
